# Predictors of seizure as presenting symptom of cerebral cavernomas

**DOI:** 10.1007/s10143-026-04246-5

**Published:** 2026-03-28

**Authors:** Carmelo Lucio Sturiale, Matteo Palermo, Maria Elena Flacco, Giorgio Mantovani, Alessio Albanese, Pasquale De Bonis, Alba Scerrati

**Affiliations:** 1 Department of Neurosurgery, Fondazione Policlinico Universitario A. Gemelli IRCCS, Università Cattolica del Sacro Cuore, Rome, Italy; 2 Environmental and Preventive Sciences, University of Ferrara, Ferrara, Italy; 3 Department of Translational Medicine, University of Ferrara, Ferrara, Italy; 4 Department of Neurosurgery, Anna University Hospital of Ferrara, SantFerrara, Italy

**Keywords:** Cavernomas, Cavernous angiomas, Epilepsy, Seizures, Epileptogenesis, Bleeding

## Abstract

Cerebral cavernous malformations (CCMs) are vascular lesions frequently presenting with seizures on presentation. Studies have widely analyzed topography as a determinant of epileptogenesis. However, the association between lesion volumetry and epileptic risk remains poorly investigated, as no objective volumetric thresholds have been established to stratify seizure risk. We conducted a multicentric case–control study including 230 adult patients with CCMs. Patients were grouped according to their initial presentation: seizure (*n* = 75) versus non-seizure (*n* = 155). We calculated the volumes of the lesions using the ABC/2 method and categorized them into quartiles. Subsequently, we run a multivariate logistic regression assessing independent predictors of epilepsy, adjusting for demographic, clinical, and radiological factors. We also estimated the diagnostic accuracy of lesion volume thresholds (> 11.9 mm^3^, > 80 mm^3^, > 300 mm^3^). Lesion volume was significantly associated with epileptic risk. Lesions ≥ 80 mm^3^ were shown to have higher odds of seizures on presentation (OR 86.4, 95% CI 9.94–751). We found hemorrhagic presentation (OR 60.3, 95% CI 6.50–558) and frontal or temporal location (OR 6.34, 95% CI 2.99–113.4) to be also independent predictors of seizure. Differently, demographic and pharmacologic factors were not independently predictive. Lesion volume, hemorrhage, andfrontal or temporal lobe location were found to be the major predictors of epileptic seizure as first manifestation. Therefore, incorporating volumetric assessment into routine MRI evaluation, after establishing absolute thresholds, may improve individualized risk stratification and guide early management decisions.

## Introduction

Cerebral cavernous malformations (CCMs) are vascular malformations composed of clusters of dilated, thin-walled capillaries lacking intervening brain parenchyma [25]. These lesions are relatively common, with an estimated prevalence of about 0.1–0.5% in the general population, and many are discovered incidentally on MRI [11]. Although often incidentally discovered, sometimes symptomatic CCMs may cause significant ictal neurologic events. Their fragile vessels, in fact, predispose to micro- or macro-hemorrhages, resulting in possible focal deficits depending on their topography; other times, however, the clinical onset may be characterized by sudden partial or generalized tonic–clonic epileptic seizures.

Among symptomatic CCMs, epileptic seizures are one of the most frequent and disabling presentations, which may occur in up to roughly 40% of patients with CCM, with some series report rates approaching 70–80% [25]. In these cases, epilepsy is the initial clinical manifestation that leads to the diagnosis of a CCM, and requires a long-term management, substantially contributing to patient morbidity [7, 25]

Several lesion characteristics are known to influence the risk of epilepsy. Lesion location is considered particularly important: CCMs involving the cerebral cortex, in fact, especially in the temporal or frontal lobes, appear highly epileptogenic. Larger lesions and those surrounded by hemosiderin-laden gliotic tissue are also thought to amplify cortical irritability. In summary, classic risk factors for CCM‐related seizures include cortical location, large lesion size/volume, and evidence of prior hemorrhage with hemosiderin deposition [13, 17, 23]. Despite these observations, the quantitative contributions of lesion size and volume to epilepsy risk remain poorly defined. Most prior studies have described demographic and radiological correlates of seizure occurrence in CCMs, but few have rigorously evaluated how CCM volume predicts seizure. In particular, no robust volumetric threshold has been established to stratify for the risk [6, 13, 24, 26]. The lack of systematic volume-based analysis means that clinicians currently have no objective cut-off values to guide individualized risk assessment.

The present study was therefore designed to address this gap. We aimed to quantify the predictive role of CCM volume for epileptic episodes as the first clinical manifestation of CCM. We also sought optimal volumetric cut-offs that maximize sensitivity and specificity for epilepsy risk. By establishing clear thresholds and clarifying how CCM volume and location influence seizure propensity, this work will facilitate more precise risk stratification and inform clinical decision-making in the management of patients with CCM.

## Methods

This isanational, multicenter case–control study combining retrospective data collection across the two participating centers. The study protocol was reviewed and approved by the Local Ethical Committee (approval ID 670/2021/Oss/AOUFe). All participating institutions adhered to standardized procedures for data collection, patient confidentiality, and ethical conduct in accordance with the Declaration of Helsinki.

### Inclusion criteria

Patients older than 18 years with either a previous or new diagnosis of CCM confirmed by neuroimaging (single CCM, multiple CCMs, or familial CCM) were included. We collected data on CCM characteristics (number, location, Zabramski classification, hemorrhagic events), prior therapies, major adverse cardiovascular events (MACE), alcohol and tobacco use, comorbidities (e.g., diabetes, obesity, hypertension), modified Rankin Scale (mRS) and Glasgow Outcome Scale (GOS) scores, and surgical treatment. All medications were recorded.

Lesion volume was calculated using an adapted version of the ABC/2 method for intracerebral hemorrhage volume measurement [15] and categorized into quartiles as follows: < 11.9 mm^3^, 11.9–79 mm^3^, 80–299 mm^3^, and > 300 mm^3^. Lesion volume was calculated by a trained investigator using initial MRI imaging, which most accurately delineates the margins of cavernous malformation. Lesion volume was calculated by a trained investigator using magnetic resonance imaging, primarily based on T2-weighted and susceptibility-weighted (SWI/T2) sequences, which most accurately delineate cavernous malformation margins. Measurements were performed blinded to clinical presentation, including seizure status, to minimize observer bias. Acute bleeding was defined by intrinsic T1 hyperintensity and/or T2/FLAIR signal changes consistent with recent hemorrhage, whereas prior hemorrhage was identified on T2-weighted or susceptibility-weighted imaging (SWI), including lesions showing a peripheral hemosiderin rim.

In the seizure group (SG), we included patients whose first clinical manifestation was an epileptic seizure. The control group (CG) included patients whose first presentation was a different clinical manifestation, either neurological deficit due to cerebral hemorrhage or headache, or an incidental finding. In patients with multiple CCMs, only the clinically significant lesion—defined as the lesion most likely responsible for seizures or other neurological dysfunction, was considered for analysis. None of the patients with incidental findings presented with multiple lesions.

### Ethical statement and patient consent

The study protocol was reviewed and approved by the Local Ethical Committee (approval ID 670/2021/Oss/AOUFe). Patient consent was not required by our institution. All participating institutions adhered to standardized procedures for data collection, patient confidentiality, and ethical conduct in accordance with the Declaration of Helsinki.

### Statistical analysis

Potential differences in the recorded demographic and clinical characteristics among patients with CCM reporting epileptic seizures, versus CCM patients without seizures, were first evaluated using chi- squared test for categorical variables, t-test and Kruskal–Wallis test for normally distributed and non-normally distributed continuous variables, respectively (Shapiro–Wilk test was used to assess the distribution of the continuous variables). The patient-based analysis was conducted considering every patient as a single statistical unit. In patients with multiple lesions, the analysis was guided by:

(i) morpho-radiological characteristics (including lesion size, topography, hemorrhagic stigmata, and proximity to eloquent cortex),
(ii) electroencephalographic (EEG) findings.
(iii) seizure semiology, including lateralizing and localizing features of the clinical events.

The potential independent predictors of seizures were then evaluated using multivariate logistic regression. Covariates were selected for inclusion in final model using a stepwise forward process with the following inclusion criteria: clinical relevance; *p*-value < 0.15 at univariate analysis; age, concomitant use of anti-platelets and b-blockers, and lesions volume forced to entry. Moreover, to reduce the potential overfitting due to the limited number of successes, the number of covariates was limited to eight in every phase of model fitting and, in the case of multiple covariates clinically relevant and significantly associated to the outcome at univariate analyses (e.g. hypertension, smoking status or presence of multiple lesions), any improvement in model performance following the inclusion of each variable was assessed from change in the AUC. The role of lesions volume was explored either in its original form, as a continuous variable, and ordinally. To identify the most informative cut-offs, lesions volume was stratified into (a) terziles, (b) quartiles and (c) quintiles, and the association between each threshold value and the likelihood of seizures was evaluated through uni- and multivariate analyses. Additionally, as above stated, any improvement in model performance was assessed from change in the AUC. Finally, we chose to include four categories of volume as dummy variables: ≤ 11.9 mm3; > 11.9 to 80 mm3; ≥ 80 to 300 mm3; > 300 mm3, as they represented the best trade-off between data accuracy and data fragmentation. In every step of multivariate model building, a minimum events-to-variable ratio of 10 was maintained, to avoid overfitting. The goodness-of-fit was checked using Hosmer–Lemeshow test, and the predictive power assessed through C-statistics (area under the Receiving Operator Curve). Standard post-estimation tests were used to check the validity of the final model, performing multicollinearity and influential observation analyses (using standardized residuals, change in Pearson and deviance chi-square).

Some of the predictors (lesions volume) showed very high odds ratios, and almost perfectly predicted the outcome. As such, in order to provide more informative data on the diagnostic accuracy of the different cut-off volumes, we estimated the potential of: (a) lesions > 11.9 mm3 (vs. ≤ 11.9 mm3); (b) lesions > 80 mm3 (vs. ≤ 80 mm3); (c) lesions > 300 mm3 (vs. ≤ 300 mm3) to predict the onset of post-treatment seizures computing summary estimates of sensitivity, specificity, positive and negative predictive values (PPV and NPV). 95% Confidence Intervals (CI) for specificity and sensitivity and for PPV and NPV were computed according to the efficient-score method, corrected for continuity, described by Newcombe. Statistical significance was defined as a two-sided p-value < 0.05, and all analyses were carried out using Stata, version 13.1 (Stata Corp., College Station, Texas, USA, 2013).

## Results

We collected data on 230 patients, including 75 patients in the SG and 155 in the CG.

No statistically significant differences were recorded between the two groups regarding sex, alcohol consumption, diabetes, mean BMI, menopause, history of MACE or trauma, anticoagulant drug use, or other pharmacological treatments (Table 1). All 75 patients presented with epileptic seizures. Based on clinical semiology, 39 patients (52%) were classified as having partial seizures, while 36 patients (48%) were classified as having generalized tonic–clonic seizures.

**Table 1:** Demography, clinical characteristics and pharmacological treatment overall and in CCM patients with versus without epileptic seizures

*Variables*	Overall sample	Seizure	No Seizure	
	(*n* = 230)	(*n* = 75)	(*n* = 155)	*p* *
Male gender, %	47.8	49.3	47.1	0.8
Mean age in years (SD)	44.5 (19.0)	**39.0 (17.5)**	47.2 (19.2)	**0.002**
Median time from diagnosis to surgery in months, (IQR)	12.7 (3.0–71.0)	**9.0 (1.2–29.0)**	17.0 (3.0–90.0)	**0.020**
Familial CCM mutations, %				
- CCM1	32.6	0.0	**35.0**	**0.005**
- CCM2	0.0	0.0	0.0	–
- CCM3	0.0	0.0	0.0	–
Smoking status, %				
- Current	7.9	**10.8**	6.4	**0.07**
- Former	9.6	0.0	**14.2**	** < 0.001**
- Never	82.5	89.2	79.4	0.3
Alcohol drinking, % ^B^	2.2	2.7	1.9	0.7
Diabetes, %	3.5	1.3	4.5	0.2
Hypertension, %	28.7	12.0	**36.8**	** < 0.001**
Mean BMI (SD)	25.3 (3.4)	25.5 (3.8)	25.2 (3.2)	0.5
	(n = 120)	(n = 38)	(n = 82)	
Menopause status, %	28.3	23.7	30.5	0.06
	(n = 78)	(n = 3)	(n = 75)	
History of MACE, %	51.3	0.0	53.3	0.07
MACE type, %	(n = 40)	(n = 0)	(n = 40)	–
- Stroke	5.0	0.0	5.0	
- AMI	2.5	0.0	2.5	
- Arrhythmias	22.5	0.0	22.5	
- DVT/pulmonary embolism	12.5	0.0	12.5	
- Other	57.2	0.0	57.2	
Anti-platelet drugs use, %	18.7	6.7	**24.5**	**0.001**
Anticoagulant drugs use, %				0.6
- None	97.4	98.7	96.8	
- TAO	1.7	1.3	1.9	
- NAO	0.9	0.0	1.3	
B-Blockers use, %	15.6	4.0	**21.3**	**0.001**
	(N = 78)	(N = 3)	(N = 75)	
Other pharmacologic treatments, %	96.2	100	96.0	0.7

*SD* Standard deviation, *IQR* Interquartile range, *ANS* Autonomic nervous system, *CNS* Central nervous system, *MACE* Major adverse cardiovascular events, *DVT* Deep vein thrombosis, *CCM* Cerebral cavernous malformation genes; ^A^More than one answer possible. ^B^Including lesions with the following anatomical locations: thalamus, basal ganglia, insular, spinal and orbital.

In the SG, mean age was significantly lower (39.0 vs 47.2 years; *p* = 0.002), with a shorter median time from diagnosis to surgery (9.0 vs 17.0 months; *p* = 0.020), absence of CCM1 mutations (0.0% vs 35.0%; *p* = 0.005), and a lower prevalence of hypertension (12.0% vs 36.8%; *p* < 0.001), antiplatelet use (6.7% vs 24.5%; *p* = 0.001), and beta-blocker use (4.0% vs 21.3%; *p* = 0.001) (Table 1). Current and former smokers were more frequent in the SG (*p* = 0.07 and *p* < 0.001, respectively), whereas no difference was observed among never-smokers (*p* = 0.3) (Table 1). Patients in the SG were less likely to be receiving beta-blocker or antiplatelet therapy.No statistically significant differences were observed between the two groups regarding Zabramski classification. Frontal (44% vs 25.8%; *p* = 0.005) and temporal (29.3% vs 16.8%; *p* = 0.03) locations were significantly more frequent in the SG, while multiple lesions were more common in the CG (44.5% vs 6.7%; *p* < 0.001) (Table 2).Higher lesion volumes were significantly associated with the SG (median volume 2100 mm^3^ vs 270 mm^3^; *p* < 0.001), as were bleeding lesions (38.7% vs 20.0%; *p* = 0.003) (Table 2).

**Table 2:** Radiological characteristics overall and in CCM patients with versus without epileptic seizures

Variables	Overall sample		Seizure	No Seizure		
	(*n*=230)		(*n*=75)	(*n*=155)		p^*^
Zabramski classification, %	(*n* = 151)		(*n* = 71)	(*n* = 80)		**0.050**
- Accidental	0.0		2.8	0.0		
- Type I	50.0		64.8	42.1		
- Type II	47.5		28.2	54.7		
- Type III	1.3		4.2	1.1		
- Type IV	1.2		0.0	2.1		
Presence of multiple lesions, %	33.2		6.7	**44.5**		** < 0.001**
Trauma-associated lesion, %	3.0		4.0	2.6		0.6
Anatomic lesion site, %						
- Frontal	31.7	**44.0**			25.8	**0.005**
- Parietal	12.6	14.7			11.6	0.5
- Temporal	20.9	**29.3**			16.8	**0.03**
- Occipital	7.4	4.0			9.0	0.2
- Brain stem	12.6	0.0			**18.7**	** < 0.001**
- Cerebellum	10.9	2.7			**14.8**	**0.006**
- Others ^B^	3.9	5.3			3.2	0.4
Lesion volume in mm^3^:						
Median volume (IQR)	800 (120–3000)	**2100 (800–5600)**			270 (90–1200)	** < 0.001**
By quartiles of volume:						
- ≤ 11.9 mm^3^	25.2	1.3			36.8	** < 0.001**
- > 11.9 to 80 mm^3^	27.0	25.3			27.7	0.7
- ≥ 80 to 300 mm^3^	24.4	34.7			19.4	0.011
- > 300 mm^3^	23.5	38.7			16.1	** < 0.001**
Bleeding lesion, %	26.7	**38.7**			20.0	**0.003**

*SD* Standard deviation, *IQR* Interquartile range; ^B^Including lesions with the following anatomical locations: thalamus, basal ganglia, and insular.

No statistically significant difference in clinical outcomes measured by mRS was found between SG and CG (*p* = 0.15).The outcome was good (0–2 points) in most patients (90.4%).Surgery was performed in 93.3% of SG patients, significantly more frequently than in the CG (58.1%; *p* < 0.001) (Table 3).

**Table 3:** Clinical outcome overall and in CCM patients with versus without epileptic seizures

Variables	Overall sample	Seizure	No Seizure	
	(*n* = 230)	(*n* = 75)	(*n* = 155)	*p* *
GOS score, %				
- 1	0.9	0.0	1.3	0.3
- 2	12.2	5.3	**15.5**	**0.03**
- 3	16.5	12.0	18.7	0.2
- 4	70.4	**82.7**	64.5	**0.005**
- 5				
mRankin Scale:				0.15
- 0	50.0	46.7	51.6	
- 1	32.6	40.0	29.0	
- 2	7.8	5.3	9.0	
- 3	7.0	5.3	7.7	
- 4	0.9	0.0	1.3	
- 5	0.9	2.7	0.0	
- 6	0.8	0.0	1.3	
Treated with surgery, %	69.6	**93.3**	58.1	** < 0.001**

*GOS* Glasgow Outcome Scale.

Multivariate analysis confirmed lesion volume as an important predictor of epilepsy. The highest odds ratio (OR 86.4; 95% CI 9.94–751; *p* < 0.001) was observed for lesions with volume between 80 and 300 mm^3^ (Table 4). Bleeding was also a strong predictive factor for epilepsy occurrence (OR 60.3; 95% CI 6.50–558; *p* < 0.001).Frontal or temporal lesion sites were confirmed as significant predictors (OR 6.34; 95% CI 2.99–113.4; *p* < 0.001).In contrast, lower age and lower use of beta-blockers and antiplatelet drugs were not confirmed as independent predictors of epilepsy (Table 4).

**Table 4:** Logistic regression model evaluating the potential predictors of epileptic seizures among subjects with cerebral cavernous malformations

	Epileptic seizures		
	%	Adj. OR (95% CI)	*p*
Age, 10-year increase	–	1.11 (0.89–1.39)	0.3
Lesion volume:			
- ≤ 11.9 mm^3^	1.7	1 (ref. cat.)	–
- > 11.9 to 80 mm^3^	30.7	**25.9 (3.16–213)**	**0.002**
- ≥ 80 to 300 mm^3^	46.4	**86.4 (9.94–751)**	** < 0.001**
- > 300 mm^3^	53.7	**63.5 (7.63–528)**	** < 0.001**
Bleeding presentation:			
- No	2.4	1 (ref. cat.)	–
- Yes	39.4	**60.3 (6.50–558)**	** < 0.001**
Frontal or temporal lesion site:			
- No	18.4	1 (ref. cat.)	–
- Yes	45.5	**6.34 (2.99–113.4)**	** < 0.001**
Anti-platelet drugs use:			
- No	37.4	1 (ref. cat.)	–
- Yes	11.6	1.73 (0.29–10.3)	0.5
B-blockers drugs use:			
- No	37.1	1 (ref. cat.)	–
- Yes	8.3	0.63 (0.30–1.63)	0.4

*Adj* Adjusted, *OR* Odds ratio, *CI* Confidence interval.

The raw % is the proportion of subjects with the outcome among the exposed and unexposed patients (e.g. the proportion of subjects with seizures among those with asymptomatic or symptomatic lesions).

Final model based upon 230 observations, with 75 successes. Area under the ROC curve = 0.88.

Diagnostic accuracy analysis of lesion volumes (Table 5) showed that a volume > 11.9 mm^3^ was aided in excluding seizure risk due to high sensitivity and negative predictive value. A volume > 80 mm^3^ provided the best balance between sensitivity and specificity. Finally, a volume > 300 mm^3^ was highly specific and had a high positive predictive value, making it useful for confirming high-risk cases.

**Table 5:** Diagnostic accuracy of each lesion volume cut-off to predict the onset of epileptic seizures: summary estimates of sensitivity, specificity, positive and negative predictive values (PPV and NPV). CI = Confidence interval

*Lesion volume*	Sensitivity % (95% CI)	Specificity % (95% CI)	PPV % (95% CI)	NPV % (95% CI)
> 11.9 mm3	98.7 (92.8–100)	36.8 (29.2–44.9)	43.0 (35.5–50.8)	98.3 (90.8–100)
> 80 mm3	73.3 (61.9–82.9)	64.5 (56.4–72.0)	50.0 (40.3–59.7)	83.3 (75.4–89.5)
> 300 mm3	38.7 (27.6–50.6)	83.9 (77.1–89.3)	53.7 (39.6–67.4)	73.9 (66.7–80.2)

## Discussion

Our results highlight several demographic and lesion-related features that significantly influence epilepsy risk in CCM patients. Univariate analysis comparing the SG and the CG showed that epileptic patients were significantly younger, despite similar sex distribution between cohorts. This reflects the current practice, as younger patients tend to present with seizures earlier in the disease course, leading to earlier diagnosis and surgical intervention. Notably, this finding shares common ground with the study by Zhang et al., which reported that age ≤ 44 years was associated with a higher risk of seizure [27].

Furthermore, the time from diagnosis to surgery is significantly shorter in the SG, supporting the idea that seizures are often the presenting symptom accelerating the time to surgical consult. Another noteworthy observation relies on the absence of familial CCM1 mutations among epileptic patients. While this protective association warrants attention, its underlying biological basis remains speculative.

Additionally, smoking status appeared to be a relevant factor: current smokers were more prevalent among patients with seizure, whereas former smokers were significantly more frequent in the control group. In contrast, hypertension was more commonly found in the CG, plausibly reflecting the older age of the cohort.

An intriguing finding emerged from our univariate analysis, as SG patients were less likely to be on beta-blockers or antiplatelet agents [1, 10, 16, 22].This pattern might suggest a protective effect, as some studies have reported that antiplatelet therapy may reduce micro- and macro-hemorrhages and the associated inflammatory cascade, while beta-blockers could indirectly modulate seizure threshold [7, 13, 17, 20, 23, 28]. On the other hand the age could be a confounding factors for this kind of association, older people are more likely to be on that kind of drugs. However, in our multivariate analysis these associations were not significant after adjusting for other variables. It appears these factors were confounded by lesion characteristics: for example, older patients and those on cardio-active medications may have more co-morbid vascular lesions but not necessarily more seizures. This is in agreement with the study by Zhang et al., as sex, obesity, cardiovascular history, or anticoagulant use did not differ between groups (epileptic vs control) [27].However, while beta-blockers and antiplatelet agents were not independent predictors in our cohort, this does not preclude potential indirect effects on lesion stability or comorbidity burden over longer follow-up, as suggested in longitudinal observational data [18].

### Volumetry

In our large multicenter cohort of 230 patients with CCMs, lesion volume emerged as a particularly important factor. Using an adapted ABC/2 method, lesion volume was stratified into quartiles [1, 2, 14]. Patients in the upper quartiles had much higher epilepsy risk: for example, lesions ≥ 80 mm^3^ had 86-fold greater odds of presenting with seizures than very small lesions (≤ 11.9 mm^3^). This suggests that larger CCMs, which likely produce a greater burden of hemosiderin and gliosis in adjacent cortex, are inherently more epileptogenic. Indeed, lesion volume may serve as a surrogate marker for the extent of chronic blood breakdown products, as prior studies have demonstrated that iron deposition increases neural irritability [15]. Clinically, this implies that quantitative volumetric assessment can enhance risk stratification: even moderate increases in CCM size markedly raise seizure risk. In fact, the logistic regression models confirmed lesion volume, symptomatic presentation, andfrontal or temporal location as independent predictors of seizures. The diagnostic accuracy analysis of volume thresholds further supports its role in risk stratification, with different cut-offs offering varying balances between sensitivity and specificity [19].

### Hemorrhagic presentation and lesion topography

Hemorrhage was a second key predictor of epilepsy in our cohort. A significantly higher proportion of SG lesions had bled (38.7% vs.20.0%, *p* = 0.003, Table 2), and on multivariate analysis bleeding conferred an OR = 60 for seizure presentation. This is biologically plausible: acute or chronic microhemorrhages release hemosiderin that can cause cortical hyperexcitability in particular in case of cortical or juxta cortical topography. Indeed, in an independent Chinese series by Zhang et al., 86% of seizures-associated CCMs showed a hemosiderin rim, and hemosiderin was a strong risk factor (OR = 16.5)[15]. Similarly, lesion location in frontal and temporal lobes was significantly over-represented in the SG (44% vs.25.8% frontal, 29.3% vs.16.8% temporal; *p* = 0.005 and *p* = 0.03 respectively): among them, the majority were also located in cortical or juxta cortical region, although this difference was not statistically significant in our series. Our multivariate model confirmed frontal/temporal site as an independent risk factor (OR = 6.34). These findings align with prior studies showing that temporal lobe CCMs are highly epileptogenic. For example, Shih et al.found that temporal lesions accounted for the majority of CCM-related epilepsy and drug-resistant epilepsy [24]. By contrast, as reported by Dulamea et al., CCMs in deep or non-eloquent regions are less likely to cause seizures [18]. Our data thus support the view that hemorrhagic, superficially located CCMs carry the greatest seizure risk. Interestingly, because anti-platelet agents and beta-blockers are more commonly prescribed in older patients, age was included in the multivariable model to control for potential confounding. After adjustment for age, neither medication class was independently associated with seizure occurrence.

### Surgical management and clinical outcomes

We observed markedly higher rates of surgical resection in the SG (93.3% vs.58.1%, *p* < 0.001).This likely reflects two factors: first, seizures themselves often prompt consideration of resection to achieve seizure control; and second, large or hemorrhagic lesions may be deemed safer or more urgent surgical targets. Notably, time to surgery was significantly shorter in SG. This indicates clinicians tended to operate sooner on seizure-presenting CCMs than on lesions found incidentally or after non-epileptic hemorrhage. Despite these management differences, the overall functional outcomes were similarly favorable in both groups (90.4% of patients had good outcome, mRS 0–2). However, it should be emphasized that our study did not rigorously collect seizure-specific outcome data, such as Engel class or long-term seizure freedom, beyond noting general disability scores. This is a limitation: Engel or ILAE outcome scales would more directly capture the impact of surgery on epilepsy control. Future studies should systematically record postoperative seizure status to correlate preoperative risk factors with surgical success.

### Clinical implications

The clinical implications of our findings are twofold. First, they highlight that large, frontal or temporal lobe locatated, hemorrhagic CCMs require vigilant monitoring for seizures. Patients with such lesions may benefit from early neurosurgical consultation or empirical antiseizure therapy [5, 8, 9], given their high risk. Second, our results suggest that incorporating volumetric analysis into routine MRI assessment could improve risk stratification: for example, a lesion exceeding 80 mm^3^ should raise an alert for epilepsy risk [3, 4, 12, 21].

**Fig. 1:**
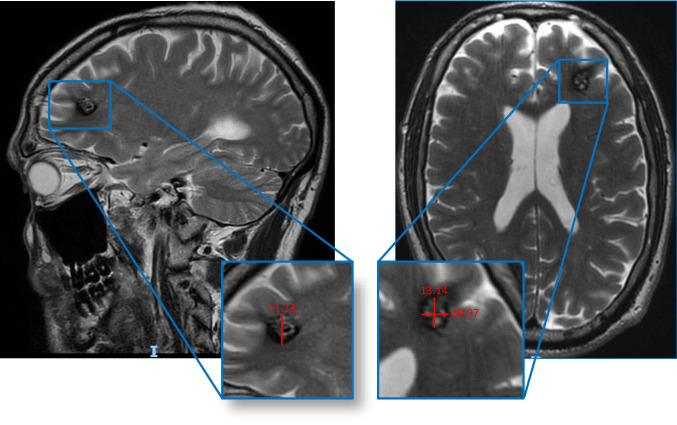
Diagnostic accuracy metrics by lesion volume cutoffs

For future research, prospective studies are needed to validate these risk thresholds and to explore biological mechanisms. Advanced imaging techniques, such as quantitative susceptibility ma.

pping (QSM) to measure iron deposition, might refine our understanding of how hemorrhagic burden correlates with epileptogenesis. Genetic and molecular studies could also determine whether certain CCM genotypes predispose more to seizures when combined with large lesion size Fig. 1.

### Strengths and limitations

A key strength of this work is its multicenter design with prospective/retrospective data from multiple Italian centers, yielding a large and diverse sample. This improves generalizability compared to single-center series. Another strength is the systematic volumetric classification of CCMs, an approach adapted from hemorrhage studies, which provided objective quantification of lesion size. Our volumetric cut-offs (≤ 11.9, 12–80, 80–299, ≥ 300 mm^3^) were data-driven and enabled us to detect the non-linear effect of size on seizure risk. Future studies should aim to establish absolute thresholds that can be incorporated into clinical counseling to improve individualized risk assessment. Measurements were not obtained using 3D volumetric segmentation, which limits the precision of volume estimation. Nonetheless, limitations exist. The study was observational and partly retrospective, which may introduce selection biases. Imaging protocols varied among centers, so volumetric data may have some measurement variability. We did not analyze some potentially relevant factors, such as presence of associated developmental venous anomalies or detailed genetic subtypes, which could influence epilepsy risk. Crucially, as noted, we did not capture long-term seizure outcomes in a standardized way, limiting our ability to link the identified predictors to postoperative seizure freedom. This limits the ability to directly translate these findings into surgical decision-making and warrants further investigation in future studies. Also, our use of GOS scores was hampered by incomplete data, so we relied on mRS for functional status.

## Conclusion

This study is the first to provide objective, data-driven volumetric thresholds for predicting seizures in CCMs, demonstrating that a lesion volume ≥ 80 mm^3^ significantly increases the risk alongside hemorrhage and frontal/temporal location, while demographic and medical treatment factors are not independently predictive.

By integrating lesion volume with frontal or temporal lobe ocation and hemorrhagic status, our findings offer a novel and more precise approach to individualized risk stratification, supporting the routine inclusion of volumetric analysis in MRI evaluations to guide early management decisions.

These findings should inform patient counseling and management, and they provide a foundation for future research into personalized risk assessment in CCM.

## Data Availability

No datasets were generated or analysed during the current study.

## Abbreviations

CCMs: Cerebral cavernous malformations
mRS: Modified Rankin Scale
GOS: Glasgow outcome scale
SG: Epilepsy group
CG: Control group
QSM: Quantitative susceptibility mapping
