# Correction to: Predictors of seizure as presenting symptom of cerebral cavernomas

**DOI:** 10.1007/s10143-026-04301-1

**Published:** 2026-04-21

**Authors:** Carmelo Lucio Sturiale, Matteo Palermo, Maria Elena Flacco, Giorgio Mantovani, Alessio Albanese, Pasquale De Bonis, Alba Scerrati

**Affiliations:** 1 Department of Neurosurgery, Fondazione Policlinico Universitario A. Gemelli IRCCS, Università Cattolica del Sacro Cuore, Rome, Italy; 2 Environmental and Preventive Sciences, University of Ferrara, Ferrara, Italy; 3 Department of Translational Medicine, University of Ferrara, Ferrara, Italy; 4 Department of Neurosurgery, Anna University Hospital of Ferrara, SantFerrara, Italy

**Correction to: Neurosurgical Review**

10.1007/s10143-026-04246-5

The authors regret that the authors’ first and last names were reversed in the original published article.

The correct author names appeared in this erratum article.

The original article can be found at 10.1007/s10143-026-04246-5.

The original article online has been corrected.

